# Supplementary material for: Hunger- and thirst-sensing neurons modulate a neuroendocrine network to coordinate sugar and water ingestion
Source: eLife. 2023 Sep 21;12:RP88143. doi: 10.7554/eLife.88143 (PMC10513480; doi:10.7554/eLife.88143)
Supplement: Supplementary file 1. — Number of synapses from the four ISNs onto different cell types, including the Flywire tracing contributions of different laboratories. [file elife-88143-supp1.docx]

|  | **Cell type / Classification** | **Flywire ID** | **Synapses from ISN 720575940619731393** | **Synapses from ISN 720575940628363855** | **Synapses from ISN 720575940625627932** | **Synapses from ISN 720575940624153528** | **Total synapses from 4 ISNs** | **Total synapses per cell type** | **Tracing contributions (number of edits)** |
| --- | --- | --- | --- | --- | --- | --- | --- | --- | --- |
| 1 | FLAa2 | 720575940627559623 | 19 | 20 | 29 | 25 | 93 |  | Jefferis Lab: Irene Salgarella (3), Varun Sane (1). |
| 2 | FLAa2 | 720575940613754833 | 19 | 32 | 21 | 19 | 91 |  | Murthy and Seung Labs: James Hebditch (1), Ben Silverman (1), remer tancontian (1). Jefferis Lab: Bhargavi Parmar (4). |
| 3 | FLAa2 | 720575940621782057 | 18 | 14 | 21 | 7 | 60 |  | Murthy and Seung Labs: Nash Hadjerol (6). |
| 4 | FLAa2 | 720575940623605180 | 10 | 10 | 14 | 14 | 48 |  | Dickson Lab: Alisa Poh (1). Murthy and Seung Labs: Austin T Burke (16), Shirleyjoy Serona (1). |
| 5 | FLAa2 | 720575940625218590 | 7 | 16 | 11 | 10 | 44 |  | Jefferis Lab: Irene Salgarella (8), Arti Yadav (1). |
| 6 | FLAa2 | 720575940620994292 | 7 |  | 23 | 14 | 44 |  | Jefferis Lab: Irene Salgarella (1), Greg Jefferis (1), Philipp Schlegel (1), Bhargavi Parmar (1). Murthy and Seung Labs: Ben Silverman (1), Kendrick Joules Vinson (15), Joshua Bañez (2). |
| 7 | FLAa2 | 720575940632134483 | 17 | 6 | 13 | 6 | 42 |  | Jefferis Lab: Imaan Tamimi (1), Arti Yadav (1). Murthy and Seung Labs: James Hebditch (2), Nash Hadjerol (1). |
| 8 | FLAa2 | 720575940619283553 | 11 | 10 | 9 | 12 | 42 |  | Jefferis Lab: Arti Yadav (1). Murthy and Seung Labs: Austin T Burke (14). |
| 9 | FLAa2 | 720575940620306300 | 21 |  | 5 | 12 | 38 |  | Murthy and Seung Labs: Austin T Burke (1), James Hebditch (2). Jefferis Lab: Arti Yadav (1). |
| 10 | FLAa2 | 720575940611991830 | 15 | 11 | 5 |  | 31 |  | Jefferis Lab: Irene Salgarella (2). Murthy and Seung Labs: Austin T Burke (11). |
| 11 | FLAa2 | 720575940626512881 | 7 |  | 14 | 10 | 31 |  | Murthy and Seung Labs: Mendell Lopez (10). |
| 12 | FLAa2 | 720575940621680264 | 8 |  | 10 | 16 | 34 |  | Jefferis Lab: Anjali Pandey (1). Murthy and Seung Labs: Austin T Burke (12), J. Anthony Ocho (3). |
| 13 | FLAa2 | 720575940637381466 | 11 | 5 |  | 12 | 28 |  | Jefferis Lab: Irene Salgarella (4). Murthy and Seung Labs: Zairene Lenizo (1). |
| 14 | FLAa2 | 720575940632695904 | 6 | 11 | 10 |  | 27 |  | Jefferis Lab: Irene Salgarella (4), Rashmita Rana (1). |
| 15 | FLAa2 | 720575940631570636 |  | 6 | 14 | 6 | 26 |  | Murthy and Seung Labs: Austin T Burke (5), Shirleyjoy Serona (3), Mendell Lopez (45). |
| 16 | FLAa2 | 720575940621605964 | 6 |  | 12 | 8 | 26 |  | Seung Lab: Zhihao Zheng (1). Jefferis Lab: Irene Salgarella (9), Márcia Santos (2). |
| 17 | FLAa2 | 720575940637151807 | 8 | 5 | 13 |  | 26 |  | Seung Lab: Zhihao Zheng (4). Jefferis Lab: A. Javier (2), Dickson Lab: Alisa Poh (3). Murthy and Seung Labs: James Hebditch (2), regine salem (1). |
| 18 | FLAa2 | 720575940622161032 | 15 | 9 |  |  | 24 |  | Jefferis Lab: Irene Salgarella (4). Murthy and Seung Labs: Nash Hadjerol (8), Joshua Bañez (14). |
| 19 | FLAa2 | 720575940612087922 | 6 |  | 12 | 6 | 24 |  | Jefferis Lab: Irene Salgarella (5). Murthy and Seung Labs: Austin T Burke (2), Nash Hadjerol (5). |
| 20 | FLAa2 | 720575940623752741 | 12 | 5 | 5 |  | 22 |  | Jefferis Lab: Irene Salgarella (5). |
| 21 | FLAa2 | 720575940643191575 |  | 6 | 15 |  | 21 |  | Murthy and Seung Labs: Mendell Lopez (11). Jefferis Lab: Arti Yadav (1). |
| 22 | FLAa2 | 720575940635191438 |  | 12 |  | 8 | 20 |  | Jefferis Lab: Irene Salgarella (3). Murthy and Seung Labs: Zairene Lenizo (2). |
| 23 | FLAa2 | 720575940610624206 | 8 |  | 12 |  | 20 |  | Jefferis Lab: Irene Salgarella (4). Murthy and Seung Labs: remer tancontian (2), J. Dolorosa (1). |
| 24 | FLAa2 | 720575940644751651 | 6 | 6 | 6 |  | 18 |  | Jefferis Lab: Arti Yadav (1). Murthy and Seung Labs: Doug Bland (19). |
| 25 | FLAa2 | 720575940623989245 |  | 7 |  | 7 | 14 |  | Jefferis Lab: Irene Salgarella (5). Murthy and Seung Labs: remer tancontian(1), Austin T Burke (1), Ben Silverman (2). |
| 26 | FLAa2 | 720575940623231527 |  |  | 9 | 5 | 14 |  | Jefferis Lab: Yijie Yin (3). Dickson Lab: Alisa Poh (1). Murthy and Seung Labs: James Hebditch (1), Mendell Lopez (6). |
| 27 | FLAa2 | 720575940604764862 | 7 |  | 6 |  | 13 |  | Jefferis Lab: Irene Salgarella (2). Murthy and Seung Labs: Mendell Lopez (1). |
| 28 | FLAa2 | 720575940628857850 |  |  | 13 |  | 13 |  | Wolf Lab: fred wolf (1). Jefferis Lab: Arti Yadav (1), Yijie Yin (4). Murthy and Seung Labs: Mendell Lopez (3), remer tancontian (1). |
| 29 | FLAa2 | 720575940638900469 | 6 |  | 6 |  | 12 |  | Jefferis Lab: Irene Salgarella (4). |
| 30 | FLAa2 | 720575940619398872 | 12 |  |  |  | 12 |  | Jefferis Lab: A. Javier (1), Chitra Nair (1). Dickson Lab: Alisa Poh (13). Murthy and Seung Labs: Zairene Lenizo (4). |
| 31 | FLAa2 | 720575940631044141 | 6 |  | 6 |  | 12 |  | Murthy and Seung Labs: James Hebditch (2). Jefferis Lab: Arti Yadav (1). |
| 32 | FLAa2 | 720575940638996413 |  | 5 | 7 |  | 12 |  | Dickson Lab: Alisa Poh (8). Murthy and Seung Labs: Kyle Patrick Willie (2), Shirleyjoy Serona (3), Kendrick Joules Vinson (1), Joshua Bañez (1), James Hebditch (1). Jefferis Lab: Arti Yadav (1), Bhargavi Parmar (1). |
| 33 | FLAa2 | 720575940624735565 | 6 |  | 5 |  | 11 |  | Dickson Lab: Alisa Poh (1). Jefferis Lab: Varun Sane (1), Arti Yadav (1). Murthy and Seung Labs: Ryan Willie (24), Rey Adrian Candilada (1). |
| 34 | FLAa2 | 720575940622266748 |  | 5 |  | 5 | 10 |  | Jefferis Lab: Rashmita Rana (1), Zeba Vohra (1). Murthy and Seung Labs: Shirleyjoy Serona (15). |
| 35 | FLAa2 | 720575940644327022 |  | 5 | 5 |  | 10 |  | Murthy and Seung Labs: James Hebditch (2). Jefferis Lab: Arti Yadav (1). Murthy and Seung Labs: J. Dolorosa (7). |
| 36 | FLAa2 | 720575940637002724 | 8 |  |  |  | 8 |  | Jefferis Lab: Imaan Tamimi (1), Irene Salgarella (5). Murthy and Seung Labs: Darrel Jay Akiatan (2), J. Anthony Ocho (18). |
| 37 | FLAa2 | 720575940612348438 |  |  |  | 8 | 8 |  | Jefferis Lab: Anjali Pandey (1). Murthy and Seung Labs: Zairene Lenizo (2), Kendrick Joules Vinson (4), Rey Adrian Candilada (3). |
| 38 | FLAa2 | 720575940623063847 | 8 |  |  |  | 8 |  | Jefferis Lab: Arti Yadav (1). Murthy and Seung Labs: Shirleyjoy Serona (17). |
| 39 | FLAa2 | 720575940616872337 |  | 7 |  |  | 7 |  | Jefferis Lab: Rashmita Rana (1). Murthy and Seung Labs: Ben Silverman (8), Kendrick Joules Vinson (6), Kyle Patrick Willie (1). |
| 40 | FLAa2 | 720575940626982165 |  | 6 |  |  | 6 |  | Seung Lab: Zhihao Zheng (4). Jefferis Lab: Irene Salgarella (4), Yijie Yin (2). Murthy and Seung Labs: Nash Hadjerol (8). |
| 41 | FLAa2 | 720575940619403435 |  | 5 |  |  | 5 |  | Jefferis Lab: Marta Costa (3), Irene Salgarella (1), Anjali Pandey (1), Varun Sane (18). |
| 42 | FLAa2 | 720575940626742532 |  |  | 5 |  | 5 |  | Jefferis Lab: Marta Costa (3), Irene Salgarella (1), Anjali Pandey (1), Varun Sane (18). |
| 43 | FLAa2 | 720575940630393427 |  |  | 5 |  | 5 |  | Jefferis Lab: Irene Salgarella (9). |
| 44 | FLAa2 | 720575940616510425 |  | 5 |  |  | 5 |  | Dickson Lab: Alisa Poh (5). Murthy and Seung Labs: Rey Adrian Candilada (1). |
| 45 | FLAa2 | 720575940634776511 |  |  | 5 |  | 5 |  | Jefferis Lab: Imaan Tamimi (2), Arti Yadav (1). Dickson Lab: Alisa Poh (2). Murthy and Seung Labs: Mendell Lopez (8), Ariel Dagohoy (19), Joshua Bañez (2), Ben Silverman (1). |
| 46 | FLAa2 | 720575940605589378 |  |  |  | 5 | 5 | 1080 | Jefferis Lab: Arti Yadav (1). Murthy and Seung Labs: Austin T Burke (15), Zairene Lenizo (1). |
| 47 | Handshake | 720575940651601910 | 54 | 51 | 51 | 35 | 191 |  | Jefferis and Waddell Labs: Joseph Hsu (1). Wolf Lab: fred wolf (1). Scott Lab: Amanda Abusaif (2). Murthy and Seung Labs: Ariel Dagohoy (2), J. Anthony Ocho (2), Shirleyjoy Serona (13). Jefferis Lab: Sangeeta Sisodiya (23). |
| 48 | Handshake | 720575940626449158 | 46 | 46 | 42 | 37 | 171 |  | Jefferis and Waddell Labs: Joseph Hsu (1). Scott Lab: Amanda Abusaif (1). |
| 49 | Handshake | 720575940618791515 | 45 | 51 | 49 | 38 | 183 |  | Wolf Lab: fred wolf (2). Scott Lab: Amanda Abusaif (3). |
| 50 | Handshake | 720575940622839786 | 39 | 56 | 44 | 38 | 177 | 722 | Jefferis and Waddell Labs: Joseph Hsu (1). Jefferis Lab: Laia Serratosa (1), Rashmita Rana (1). Dickson Lab: Alisa Poh (1). Murthy and Seung Labs: Kyle Patrick Willie (4), remer tancontian (5), Austin T Burke (8). Itisha Joshi (15). |
| 51 | Cowboy L fragment | 720575940611730674 | 72 | 95 | 75 | 59 | 301 |  | Simpson Lab: Li Guo (7). Jefferis Lab: Yijie Yin (2), Siqi Fang (3). Scott Lab: Amanda González-Segarra (7), Amanda Abusaif (1). Murthy and Seung Labs: J. Anthony Ocho (2), Mendell Lopez (1), Shaina Mae Monungolh (7), Ben Silverman (1), Nash Hadjerol (1), remer tancontian (1), Miguel Albero (2), Rey Adrian Candilada (2). |
| 52 | Cowboy R | 720575940624514492 | 36 | 51 | 38 | 34 | 159 | 460 | Jefferis and Wilson Labs: Laia Serratosa Capdevila (6). Jefferis Lab: Yijie Yin (6), Katharina Eichler (2), Zeba Vohra (18). Scott Lab: Amanda Abusaif (6). Murthy and Seung Labs: J. Dolorosa (1), Austin T Burke (10), Darrel Jay Akiatan (13) |
| 53 | SEZ: GNG.GNG.2607 | 720575940633170969 | 37 | 30 | 33 | 24 | 124 |  | Murthy and Seung Labs: Ryan Willie (32), Joshua Bañez (1), remer tancontian (1), Nash Hadjerol (1). Jefferis Lab: Katharina Eichler (1), Dhwani Patel (4), Griffin Badalemente (2), Dharini Sapkal (1). |
| 54 | SEZ: PRW.GNG.9 | 720575940616167730 | 35 | 19 | 40 | 21 | 115 |  | Jefferis Lab: Arti Yadav (1), Griffin Badalemente (2), Dharini Sapkal (5), Yashvi Patel (2), Bhargavi Parmar (1). Scott Lab: Amanda González-Segarra (3). Murthy and Seung Lab: Mendell Lopez (12). |
| 55 | SEZ: PRW.PRW.166 | 720575940622732253 | 5 | 12 | 17 |  | 34 |  | Jefferis Lab: Rashmita Rana (1), Zeba Vohra (4). Murthy and Seung Labs: Shirleyjoy Serona (55), Austin T Burke (1). Kim Lab: Chan Hyuk Kang (1). |
| 56 | SEZ: PRW.PRW.214 | 720575940625178768 | 8 | 6 |  | 17 | 31 |  | Jefferis Lab: Rashmita Rana (1), Sangeeta Sisodiya (1). Murthy and Seung Labs: Ben Silverman (7), Shirleyjoy Serona (23), Austin T Burke (1), Zairene Lenizo (4), Kyle Patrick Willie (2). |
| 57 | SEZ: PRW.GNG.6 | 720575940614736290 | 8 | 8 | 5 | 5 | 26 |  | Murthy and Seung Labs: Ryan Willie (1), Ben Silverman (1), J. Anthony Ocho (37), Nash Hadjerol (2). Jefferis Lab: Chitra Nair (1), Yashvi Patel (1). |
| 58 | SEZ: PRW.GNG.12 | 720575940619042427 |  |  | 8 |  | 8 |  | Seeds Hampel Lab: Patricia Pujols (7). Murthy and Seung Labs: Zairene Lenizo (4), Rey Adrian Candilada (1), remer tancontian (1), Kendrick Joules Vinson (1). Jefferis Lab: Bhargavi Parmar (5). |
| 59 | SEZ: PRW.PRW.146 | 720575940621490721 |  |  |  | 6 | 6 |  | Jefferis Lab: Arti Yadav (1). Murthy and Seung Labs: J. Anthony Ocho (2), Ben Silverman (3), Shaina Mae Monungolh (1), Kyle Patrick Willie (4). |
| 60 | SEZ: GNG.GNG.1075 | 720575940620627803 | 5 |  |  |  | 5 |  | Jefferis Lab: Shanice Bailey (3), Rashmita Rana (1), Dhwani Patel (1). Murthy and Seung Labs: J. Anthony Ocho (1). |
| 61 | SEZ: PRW.PRW.42 | 720575940613120721 |  |  | 5 |  | 5 |  | Murthy and Seung Labs: Nash Hadjerol (9). Kim Lab: hanetwo (1). |
| 62 | SEZ: PRW.PRW.62 | 720575940615052812 |  |  | 5 |  | 5 |  | Jefferis Lab: Arti Yadav (1). Murthy and Seung Labs: Austin T Burke (1), Zairene Lenizo (2), Rey Adrian Candilada (1), Szi-chieh Yu (1). Jefferis Lab: Dharini Sapkal (1). |
| 63 | SEZ: PRW.PRW.199 | 720575940624532408 |  |  | 5 |  | 5 |  | Jefferis Lab: Arti Yadav (1). Murthy and Seung Labs: Jay Gager (9). Itisha Joshi (2). |
| 64 | SEZ: FLA_L.FLA_L.47 | 720575940631299602 |  |  | 5 |  | 5 |  | Jefferis Lab: Katharina Eichler (10), Laia Serratosa (1), Marina Gkantia (11), Bhargavi Parmar (28), Dharini Sapkal (10). Murthy and Seung Labs: J. Anthony Ocho (1), Zairene Lenizo (4), Shirleyjoy Serona (41), Darrel Jay Akiatan (1). Scott Lab: Amanda Abusaif (2). Itisha Joshi (5). |
| 65 | SEZ: PRW.PRW.120 | 720575940619866059 |  |  | 5 |  | 5 |  | Jefferis Lab: Yijie Yin (1), Rashmita Rana (1), Zeba Vohra (10). Murthy and Seung Labs: Michelle Pantujan (1), Zairene Lenizo (1), regine salem (88), Rey Adrian Candilada (1), remer tancontian (3) |
| 66 | SEZ: TRdm | 720575940612921571 |  |  | 5 |  | 5 | 379 | Murthy and Seung Labs: Doug Bland (5). Jefferis Lab: Imaan Tamimi (1), Laia Serratosa (1), Griffin Badalemente (1), Dhwani Patel (15). |
| 67 | DSOG 1 | 720575940617291323 | 32 | 33 | 36 | 22 | 123 |  | Seeds Hampel Lab: Katharina Eichler (10). Jefferis and Wilson Labs: Laia Serratosa Capdevila (2). Jefferis Lab: A. Javier (8), Katharina Eichler (1), Yijie Yin (1), Dharini Sapkal (47), Arzoo Diwan (6), Dhara Kakadiya (7), Zeba Vohra (9), Dhwani Patel (10), Yashvi Patel (1). Scott Lab: Amanda Abusaif (21). Pankratz Lab: Damian Demarest (1). Murthy and Seung Labs: remer tancontian (6), J. Dolorosa (54), Kendrick Joules Vinson (3), Shaina Mae Monungolh (2), Zairene Lenizo (2). Itisha Joshi (5) |
| 68 | DSOG 1 | 720575940623529610 | 28 | 27 | 32 | 31 | 118 |  | Seeds Hampel Lab: Katharina Eichler (3). Jefferis and Wilson Labs: Laia Serratosa Capdevila (8). Jefferis Lab: Irene Salgarella (3), Yijie Yin (2), Zeba Vohra (7), Arti Yadav (16), Bhargavi Parmar (25), Chitra Nair (39), Dhara Kakadiya (4). Scott Lab: Amanda Abusaif (12). Murthy and Seung Labs: Celia D (1), remer tancontian (2), Austin T Burke (4), Zairene Lenizo (11), Shaina Mae Monungolh (26), Rey Adrian Candilada (1), Shirleyjoy Serona (24), J. Anthony Ocho (3). Itisha Joshi (6) |
| 69 | DSOG 1 | 720575940623338281 | 19 | 14 | 8 | 8 | 49 |  | Seeds Hampel Lab: Katharina Eichler (8). Jefferis Lab: Siqi Fang (14), Katharina Eichler (4), Irene Salgarella (2), Griffin Badalemente (7), Nidhi Patel (3), Zeba Vohra (15), Dhwani Patel (10), Chitra Nair (8), Yashvi Patel (15), Dharini Sapkal (19), Arti Yadav (4). Murthy and Seung Labs: Rey Adrian Candilada (6), Shaina Mae Monungolh (45). Kim Lab: Chan Hyuk Kang (2). Scott Lab: Amanda Abusaif (12). |
| 70 | DSOG 1 | 720575940644666660 | 13 | 5 | 17 | 5 | 40 | 330 | Seeds Hampel Lab: Katharina Eichler, Steven Calle, Lucia Kmecova, Alexis E Santana Cruz. Murthy and Seung Labs: remer tancontian, Zairene Lenizo. |
| 71 | SEZ & SMP: FLA_L.SMP_L.27 | 720575940626500362 | 19 | 5 | 18 | 8 | 50 |  | Jefferis Lab: Greg Jefferis (2). Dickson Lab: Alisa Poh (1). Murthy and Seung Labs: Shirleyjoy Serona (25), regine salem (17), Mendell Lopez (1), Shaina Mae Monungolh (2). |
| 72 | SEZ & SMP: VESa1 | 720575940632951597 | 12 |  | 19 | 16 | 47 |  | Murthy and Seung Labs: Austin T Burke (72), Sarah Morejohn (1), Kyle Patrick Willie (11), James Hebditch (6), Doug Bland (5), Nash Hadjerol (39), Ben Silverman (7), J. Dolorosa (3), Zairene Lenizo (48), Mendell Lopez (9), Shaina Mae Monungolh (5), Rey Adrian Candilada (7), regine salem (80), remer tancontian (19), Darrel Jay Akiatan (13), Joshua Bañez (112), Ariel Dagohoy (9), Kendrick Joules Vinson (27), Miguel Albero (4), Shirleyjoy Serona (89), Michelle Pantujan (14), J. Anthony Ocho (11). Jefferis and Wilson Labs: Laia Serratosa Capdevila (6). Jefferis Lab: Marlon Blanquart (1), Imaan Tamimi (5), Yijie Yin (3), Irene Salgarella (3), Varun Sane (4), Griffin Badalemente (21), Philipp Schlegel (9), Dharini Sapkal (35), Chitra Nair (34), Arzoo Diwan (8), Zeba Vohra (24), Anjali Pandey (2), Dhara Kakadiya (48), Bhargavi Parmar (10), Kaushik Parmar (1), Arti Yadav (10), Yashvi Patel (12), Greg Jefferis (1). Janelia Tracers: Tansy Yang (38). Selcho Lab: Mareike Selcho (1). |
| 73 | SEZ & SMP: FLA_L.SMP_L.32 | 720575940627436554 | 12 | 16 | 6 | 12 | 46 |  | Jefferis Lab: Greg Jefferis (1), Christophe Dunne (1), Bhargavi Parmar (1). Murthy and Seung Labs: Mendell Lopez (1), Joshua Bañez (2). |
| 74 | SEZ & SMP: FLA_R.SMP_R.32 | 720575940645920052 | 7 |  | 17 | 13 | 37 |  | Jefferis and Wilson Labs: Laia Serratosa Capdevila (1). Wolf Lab: fred wolf (1). Huetteroth Lab: Wolf Huetteroth (1). Murthy and Seung Labs: Zairene Lenizo (6), Shirleyjoy Serona (29), Ariel Dagohoy (28), Shaina Mae Monungolh (11). Jefferis Lab: Márcia Santos (1). |
| 75 | SEZ & SMP: BiT2 | 720575940621662332 | 7 | 7 | 14 | 8 | 36 |  | Murthy and Seung Labs: Austin T Burke (47), Joshua Bañez (9), Kyle Patrick Willie (16). Jefferis Lab: Imaan Tamimi (12), Yijie Yin (2), Bhargavi Parmar (2), Dharini Sapkal (1). Scott Lab: Amanda Abusaif (14). |
| 76 | SEZ & SMP: FLA_R.SMP_R.30 | 720575940637780969 | 5 | 5 | 10 | 14 | 34 |  | Jefferis Lab: Arti Yadav (1), Dhwani Patel (5). Murthy and Seung Labs: Shirleyjoy Serona (3), Ryan Willie (2), Kyle Patrick Willie (2), Rey Adrian Candilada (1). |
| 77 | SEZ & SMP: Lgr3/FLAa3 | 720575940626452879 | 8 | 15 | 9 |  | 32 |  | Jefferis Lab: Yijie Yin (1), Anjali Pandey (1). Murthy and Seung Labs: James Hebditch (1), Mendell Lopez (21), Austin T Burke (3), Joshua Bañez (3), Kendrick Joules Vinson (4). |
| 78 | SEZ & SMP: SMPpv2 | 720575940623701768 |  | 6 | 9 |  | 15 |  | Jefferis Lab: Greg Jefferis (1), Yijie Yin (2), A. Javier (3), Irene Salgarella (5). Scott Lab: Amanda Abusaif (1). Murthy and Seung Labs: Szi-chieh Yu (15), Zairene Lenizo (9). |
| 79 | SEZ & SMP: SMPpv2 | 720575940628310275 |  | 7 |  | 5 | 12 |  | Jefferis and Wilson Labs: Laia Serratosa Capdevila (9). Jefferis Lab: Katharina Eichler (9), Imaan Tamimi (5), Irene Salgarella (4), Philipp Schlegel (39). Murthy and Seung Labs: James Hebditch (1), Rey Adrian Candilada (10). |
| 80 | SEZ & SMP: Lgr3/FLAa3 | 720575940629764906 |  | 5 |  |  | 5 |  | Jefferis and Waddell Labs: Joseph Hsu (3). Murthy and Seung Labs: Austin T Burke (14). Jefferis Lab: Anjali Pandey (1). |
| 81 | SEZ & SMP: Gallinule | 720575940618926757 |  |  |  | 5 | 5 | 319 | Jefferis and Waddell Labs: Joseph Hsu (8). Jefferis Lab: Yijie Yin (4), Imaan Tamimi (1), Rashmita Rana (2). Murthy and Seung Labs: Mendell Lopez (2). |
| 82 | BiT | 720575940610708430 | 71 | 74 | 93 | 63 | 301 | 301 | Jefferis Lab: Yijie Yin (3), A. Javier (1), Imaan Tamimi (22), Varun Sane (2), Griffin Badalemente (1), Dhara Kakadiya (1), Arti Yadav (6). Murthy and Seung Labs: Claire McKellar (1), Michelle Pantujan (2), Austin T Burke (2), James Hebditch (1), J. Anthony Ocho (420), Rey Adrian Candilada (1), Nash Hadjerol (4). Wes Murfin (1). Scott Lab: Amanda Abusaif (30). |
| 83 | Ascending neuron: FLA_R.PRW.5 | 720575940625841241 | 20 | 6 | 16 | 27 | 69 |  | Seeds Hampel Lab: Steven Calle (1). Jefferis Lab: Katharina Eichler (8). Kim Lab: Chan Hyuk Kang (2). Murthy and Seung Labs: M Sorek (2), J. Anthony Ocho (1), Zairene Lenizo (1). |
| 84 | Ascending neurons: PRW.FLA_L.15 | 720575940633548128 |  | 29 | 33 | 5 | 67 |  | Jefferis and Wilson Labs: Laia Serratosa Capdevila (11). Scott Lab: Zepeng Yao (1), Amanda Abusaif (2), Amanda González-Segarra (5), Rey Adrian Candilada (4). |
| 85 | Ascending neuron: FLA_L.FLA_L.25 | 720575940621599741 | 19 | 18 |  | 5 | 42 |  | Jefferis and Waddell Labs: Joseph Hsu (3). Jefferis and Wilson Labs: Laia Serratosa Capdevila (3). |
| 86 | Ascending neuron: PRW.SMP_L.30 | 720575940635527092 | 8 | 5 | 14 | 11 | 38 |  | Seeds Hampel Lab: Katharina Eichler (27). Jefferis and Wilson Labs: Laia Serratosa Capdevila (15). Jefferis Lab: Katharina Eichler (1), Varun Sane (1), Irene Salgarella (1), Bhargavi Parmar (10), Chitra Nair (40), Sangeeta Sisodiya (18), Zeba Vohra (4), Bhargavi Parmar (10), Dharini Sapkal (1). Murthy and Seung Labs: Zairene Lenizo (5), J. Anthony Ocho (3), Nash Hadjerol (5), Mendell Lopez (3), Joshua Bañez (4), regine salem (1), Rey Adrian Candilada (1), Kendrick Joules Vinson (3), Ariel Dagohoy (8), Kyle Patrick Willie (4), Doug Bland (31), Ryan Willie (1). Itisha Joshi (25) |
| 87 | Ascending neuron: FLA_R.PRW.2 | 720575940611548273 | 10 | 11 | 5 |  | 26 |  | Jefferis and Wilson Labs: Laia Serratosa Capdevila (11). Murthy and Seung Labs: Mendell Lopez (1), Zairene Lenizo (2), Kyle Patrick Willie (4). |
| 88 | Ascending neuron: PRW.PRW.304 | 720575940631295506 | 7 |  | 9 |  | 16 |  | Jefferis and Wilson Labs: Laia Serratosa Capdevila (3). Murthy and Seung Labs: Shaina Mae Monungolh (2), Nash Hadjerol (7). Jefferis Lab: Dhwani Patel (1). |
| 89 | Ascending neuron: PRW.PRW.343 | 720575940636263543 | 5 |  | 8 |  | 13 |  | Seung Lab: Zhihao Zheng (1). Jefferis Lab: Katharina Eichler (1), Dharini Sapkal (5). Murthy and Seung Labs: Michelle Pantujan (2), Nash Hadjerol (7), Shaina Mae Monungolh (1). Dickson Lab: Alisa Poh (2). |
| 90 | Ascending neuron: PRW.PRW.137 | 720575940620833901 |  |  | 6 | 6 | 12 |  | Murthy and Seung Labs: James Hebditch (9), Austin T Burke (11), Ben Silverman (1), Nash Hadjerol (3), Mendell Lopez (18), Ryan Willie (1), regine salem (3), Joshua Bañez (6), Rey Adrian Candilada (5), Zairene Lenizo (7), Darrel Jay Akiatan (3). Jefferis and Wilson Labs: Laia Serratosa Capdevila (3). Jefferis Lab: Katharina Eichler (10), Sangeeta Sisodiya (1), Dharini Sapkal (1), Bhargavi Parmar (37), Chitra Nair (17). Seeds Hampel Lab: Alexis E Santana Cruz (1). |
| 91 | Ascending neuron: PRW.PRW.247 | 720575940627304424 |  |  |  | 10 | 10 |  | Jefferis Lab: Dharini Sapkal (1). Murthy and Seung Labs: Shirleyjoy Serona (16), Zairene Lenizo (1), Rey Adrian Candilada (3). |
| 92 | Ascending neuron: FLA_L.GNG.5 | 720575940617662950 |  |  | 6 |  | 6 | 299 | Jefferis Lab: Katharina Eichler. Murthy and Seung Labs: Ben Silverman. |
| 93 | Descending neuron: PRW.GNG.16 | 720575940621328048 | 7 | 16 | 15 | 9 | 47 |  | Jefferis and Wilson Labs: Laia Serratosa Capdevila (3). Jefferis Lab: Katharina Eichler (1), Arti Yadav (1), Dharini Sapkal (1), Dhwani Patel (1), Bhargavi Parmar (31). Murthy and Seung Labs: Austin T Burke (1), Mendell Lopez (1), Shaina Mae Monungolh (7), Ariel Dagohoy (1). Itisha Joshi (5). |
| 94 | Descending neuron: FLA_L.GNG.3 | 720575940610610841 | 9 |  |  | 5 | 14 |  | Jefferis and Wilson Labs: Laia Serratosa Capdevila (14). Jefferis Lab: A. Javier (9), Yijie Yin (3), Katharina Eichler (1), Rashmita Rana (1). |
| 95 | Descending neuron: FLA_L.NO_OUT.7 | 720575940625587325 | 7 |  | 5 |  | 12 |  | Jefferis Lab: Philipp Schlegel (1), Katharina Eichler (15), Paul Brooks (45). Jefferis and Wilson Labs: Laia Serratosa Capdevila (3). Selcho Lab: Mareike Selcho (7). Kim Lab: Usb (1). |
| 96 | Descending neuron: GNG.GNG.1079 | 720575940620668609 |  |  | 12 |  | 12 |  | Seeds Hampel Lab: Katharina Eichler (2), Stefanie Hampel (1). Jefferis and Wilson Labs: Laia Serratosa Capdevila (1). Jefferis Lab: Griffin Badalemente (1), Dhwani Patel (30), Dharini Sapkal (18). Murthy and Seung Labs: regine salem (1). |
| 97 | Descending Neuron: Gumdrop | 720575940630697078 | 5 |  |  | 6 | 11 |  | Jefferis and Wilson Labs: Laia Serratosa Capdevila (21). Jefferis Lab: Katharina Eichler (30), Imaan Tamimi (1), Yashvi Patel (5), Arti Yadav (10), Zeba Vohra (23), Bhargavi Parmar (15). Murthy and Seung Labs: Nash Hadjerol (5). Itisha Joshi (8). |
| 98 | Descending neuron: FLA_L.NO_OUT.5 | 720575940619576001 | 6 |  | 5 |  | 11 |  | Jefferis and Wilson Labs: Laia Serratosa Capdevila (3). Jefferis Lab: Katharina Eichler (17), Paul Brooks (28), Imaan Tamimi (1), Yijie Yin (8). Murthy and Seung Labs: Michelle Pantujan (1), J. Dolorosa (1), Ben Silverman (14). |
| 99 | Descending neuron | 720575940644812398 |  | 6 | 5 |  | 11 |  | Jefferis and Wilson Labs: Laia Serratosa Capdevila (4). Jefferis Lab: A. Javier (24), Katharina Eichler (4), Rashmita Rana (1), Varun Sane (1). Selcho Lab: Mareike Selcho (8). Murthy and Seung Labs: Joshua Bañez (1). |
| 100 | Descending neuron: GNG.GNG.391 | 720575940612692889 | 6 |  |  |  | 6 | 124 | Jefferis and Wilson Labs: Laia Serratosa Capdevila (2). Jefferis Lab: Katharina Eichler (2), Zeba Vohra (1), Yashvi Patel (2). Murthy and Seung Labs: Shirleyjoy Serona (253), Darrel Jay Akiatan (5). |
| 101 | ISN | 720575940625627932 | 5 | 6 |  | 5 | 16 |  | Scott Lab: Alexander Edward Del Toro BSc (1). Jefferis Lab: Arti Yadav (1). Kim Lab: Hyungjun Choi (183), Chan Hyuk Kang (26), hanetwo (5). Murthy and Seung Labs: Ariel Dagohoy (2), Rey Adrian Candilada (3). |
| 102 | ISN | 720575940619731393 |  | 5 |  | 5 | 10 |  | Jefferis Lab: Katharina Eichler (1). Murthy and Seung Labs: Claire McKellar (1), Nash Hadjerol (2), Celia D (1), regine salem (332), Joshua Bañez (286), Kendrick Joules Vinson (8), J. Anthony Ocho (1). Scott Lab: Amanda González-Segarra (3), Alexander Edward Del Toro BSc (1). Kim Lab: Keehyun Park (6), hanetwo (19). |
| 103 | ISN | 720575940628363855 |  |  | 5 |  | 5 |  | Kim Group: Hyungjun Choi (29), Chan Hyuk Kang (25), hanetwo (3). Murthy and Seung Labs: Shaina Mae Monungolh (5), Joshua Bañez (1), Shirleyjoy Serona (126), Zairene Lenizo (1). |
| 104 | ISN | 720575940624153528 |  |  | 5 |  | 5 | 20 | Scott Lab: Amanda González-Segarra (1). Jefferis Lab: Anjali Pandey (1), Varun Sane (1). Kim Lab: Keehyun Park (1), hanetwo (22). Murthy and Seung Labs: Nash Hadjerol (12), Zairene Lenizo (4), remer tancontian (1). |
|  |  |  |  |  |  |  |  | 4034 |  |
