# Supplementary material for: Hunger- and thirst-sensing neurons modulate a neuroendocrine network to coordinate sugar and water ingestion
Source: eLife. 2023 Sep 21;12:RP88143. doi: 10.7554/eLife.88143 (PMC10513480; doi:10.7554/eLife.88143)
Supplement: Supplementary file 2. — Number of synapses from BiT onto different cell types, including the Flywire tracing contributions of different laboratories. [file elife-88143-supp2.docx]

|  | **Name** | **Neuron ID** | **Synapses from BiT** | **Total synapses from BiT per cell type** | **Tracing contributions (number of edits)** |
| --- | --- | --- | --- | --- | --- |
| 1 | IPC | 720575940650527222 | 54 |  | Jefferis Lab: Imaan Tamimi (2), Anjali Pandey (1). Murthy and Seung Labs: Austin T Burke (4). |
| 2 | IPC | 720575940603765280 | 44 |  | Maimon Lab: Gaby Maimon (1). Jefferis Lab: Katharina Eichler (2). Murthy and Seung Labs: Austin T Burke (3). |
| 3 | IPC | 720575940620932045 | 39 |  | Jefferis Lab: Imaan Tamimi (2), Laia Serratosa (7). Murthy and Seung Labs: Austin T Burke (6). |
| 4 | IPC | 720575940612923390 | 33 |  | Jefferis Lab: Imaan Tamimi (1), Laia Serratosa (6), Markus Pleijzier (1), Rashmita Rana (1). Murthy and Seung Labs: Ryan Willie (1). |
| 5 | IPC | 720575940625379859 | 30 |  | Jefferis Lab: A. Javier (4), Irene Salgarella (2), Laia Serratosa (9). Murthy and Seung Labs: Austin T Burke (4). |
| 6 | IPC | 720575940622897639 | 28 |  | Seung Lab: Zhihao Zheng (1), Jefferis Lab: A. Javier (1). Dacks Lab: Andrew Dacks (1). Murthy and Seung Labs: Austin T Burke (7). |
| 7 | IPC | 720575940643539566 | 26 |  | Jefferis Lab: Imaan Tamimi (3), A. Javier (7), Laia Serratosa (4), Rashmita Rana (1). Murthy and Seung Labs: Austin T Burke (13). |
| 8 | IPC | 720575940620628957 | 25 |  | Jefferis Lab: Imaan Tamimi (1), A. Javier (6). Murthy and Seung Labs: Austin T Burke. (18) |
| 9 | IPC | 720575940623081400 | 22 |  | Jefferis Lab: Markus Pleijzier (5). Murthy and Seung Labs: Austin T Burke (18). |
| 10 | IPC | 720575940631884883 | 22 |  | Dacks Lab: Andrew Dacks (1). Jefferis Lab: Imaan Tamimi (1), Laia Serratosa (8). Murthy and Seung Labs: Austin T Burke (7). Itisha Joshi (2). |
| 11 | IPC | 720575940628363820 | 22 |  | Jefferis Lab: Imaan Tamimi (1). Dacks Lab: Andrew Dacks (1). Murthy and Seung Labs: Austin T Burke (6). Scott Lab: Meghan Laturney (1). |
| 12 | IPC | 720575940624064295 | 22 |  | Dacks Lab: Andrew Dacks (1). Jefferis Lab: Imaan Tamimi (3), Laia Serratosa (10), Anjali Pandey (1). Murthy and Seung Labs: Austin T Burke (5). |
| 13 | IPC | 720575940628199802 | 21 |  | Jefferis Lab: Markus Pleijzier (1), A. Javier (1), Laia Serratosa (9), Philipp Schlegel (1). Murthy and Seung Labs: Austin T Burke (1). Wolf Lab: Fred Wolf (1). |
| 14 | IPC | 720575940623586284 | 17 |  | Jefferis Lab: Imaan Tamimi (1), Márcia Santos (4). Murthy and Seung Labs: Austin T Burke (66). |
| 15 | IPC | 720575940618694827 | 14 |  | Jefferis Lab: A. Javier (3), Imaan Tamimi (1), Laia Serratosa (12), Anjali Pandey (1). |
| 16 | IPC | 720575940614623455 | 10 |  | Murthy and Seung Labs: James Hebditch (4), Nash Hadjerol (1), Doug Bland (6), Joshua Bañez (6). Jefferis Lab: Philipp Schlegel (1). |
| 17 | IPC | 720575940611254681 | 7 |  | Jefferis Lab: Markus Pleijzier (2), A. Javier (3), Imaan Tamimi (9). Murthy and Seung Labs: Austin T Burke (13). Kim Lab: Minsik Yun (2). |
| 18 | IPC | 720575940615911380 | 6 | 442 | Jefferis Lab: A. Javier (6), Laia Serratosa (6), Philipp Schlegel (1). Murthy and Seung Labs: Ryan Willie (1). |
| 19 | Lgr3/FLAa3 | 720575940626983185 | 36 |  | Jefferis Lab: Irene Salgarella (10). Murthy and Seung Labs: James Hebditch (5), Kyle Patrick Willie (4), Rey Adrian Candilada (1), Kendrick Joules Vinson (11), Zairene Lenizo (2), Nash Hadjerol (4). |
| 20 | Lgr3/FLAa3 | 720575940620201084 | 32 |  | Jefferis Lab: Irene Salgarella (7), Laia Serratosa (4). Murthy and Seung Labs: James Hebditch (4). |
| 21 | Lgr3/FLAa3 | 720575940613895934 | 31 |  | Jefferis Lab: Irene Salgarella (10), Dharini Sapkal (2), Dhwani Patel (12). Murthy and Seung Labs: James Hebditch (2), Zairene Lenizo (2), Joshua Bañez (8). |
| 22 | Lgr3/FLAa3 | 720575940629696763 | 30 |  | Jefferis Lab: Anjali Pandey (1). Murthy and Seung Labs: James Hebditch (4), Nash Hadjerol (6), Ben Silverman (1), Darrel Jay Akiatan (2). |
| 23 | Lgr3/FLAa3 | 720575940644053271 | 19 |  | Jefferis Lab: Anjali Pandey (1), Christopher Dunne (11). Murthy and Seung Labs: Zairene Lenizo (1). |
| 24 | Lgr3/FLAa3 | 720575940630329903 | 19 |  | Jefferis Lab: Irene Salgarella (15). Murthy and Seung Labs: James Hebditch (3). |
| 25 | Lgr3/FLAa3 | 720575940616987426 | 19 |  | Murthy and Seung Labs: James Hebditch (4). Jefferis Lab: Anjali Pandey (8), Christopher Dunne (4), Sangeeta Sisodiya (1), Dharini Sapkal (4). |
| 26 | Lgr3/FLAa3 | 720575940639420032 | 19 |  | Jefferis Lab: Irene Salgarella (16). Murthy and Seung Labs: James Hebditch (4), Zairene Lenizo (1). |
| 27 | Lgr3/FLAa3 | 720575940614714299 | 18 |  | Jefferis Lab: Irene Salgarella (7). Murthy and Seung Labs: Nash Hadjerol (1). |
| 28 | Lgr3/FLAa3 | 720575940632879842 | 18 |  | Scott Lab: Zepeng Yao (17). Jefferis Lab: Arti Yadav (1), Bhargavi Parmar (6). Murthy and Seung Labs: J. Anthony Ocho (1), Austin T Burke (1), Zairene Lenizo (2). |
| 29 | Lgr3/FLAa3 | 720575940626179658 | 15 |  | Jefferis Lab: Irene Salgarella (10). |
| 30 | Lgr3/FLAa3 | 720575940626327070 | 15 | 271 | Murthy and Seung Labs: Austin T Burke (1), James Hebditch (4), Ariel Dagohoy (4), Nash Hadjerol (2). Janelia tracers: Tansy Yang (2). Jefferis Lab: Irene Salgarella (8), Laia Serratosa (3). Scott Lab: Zepeng Yao (3). Pankratz Lab: Damian Demarest (2). |
| 31 | SMP & SLP: SMP_L.SMP_L.276 | 720575940617650203 | 45 |  | Jefferis and Waddell Labs: Joseph Hsu (2). Murthy and Seung Labs: Kyle Patrick Willie (32). Jefferis Lab: Laia Serratosa (1), Anjali Pandey (1), Yijie Yin (4). |
| 32 | SMP & SLP: SMP_R.SMP_R.278 | 720575940619352198 | 44 |  | Murthy Lab: Lucas Encarnacion-Rivera (1), Bock Lab: Davi Bock (4). Jefferis Lab: Yijie Yin (13), Greg Jefferis (15). |
| 33 | SMP & SLP: SMPpv1; right | 720575940631373869 | 38 |  | Jefferis and Waddell Labs: Joseph Hsu (2). Jefferis Lab: Irene Salgarella (1). Murthy and Seung Labs: J. Dolorosa (2), Kyle Patrick Willie (4). |
| 34 | SMP & SLP: SMPpv1; left | 720575940633754292 | 26 |  | Jefferis Lab: Yijie Yin (2). Murthy and Seung Labs: Ben Silverman (1). |
| 35 | SMP & SLP: SMP_R.SMP_R.808 | 720575940635354981 | 12 |  | Murthy and Seung Labs: Zairene Lenizo (1), Nash Hadjerol (24), remer tancontian (5). Jefferis Lab: Anjali Pandey (1). |
| 36 | SMP & SLP: SMPpd1; left | 720575940627327750 | 12 |  | Jefferis Lab: Rashmita Rana (1). Murthy and Seung Labs: James Hebditch (3), remer tancontian (1). |
| 37 | SMP & SLP: SMP_R.SMP_R.70 | 720575940611332722 | 11 |  | Jefferis Lab: Yijie Yin (1), Rashmita Rana (1). Murthy and Seung Labs: Kyle Patrick Willie (10. |
| 38 | SMP & SLP: SMPpd1; left | 720575940619099558 | 10 |  | Jefferis Lab: A. Javier (2), Arti Yadav (1). |
| 39 | SMP & SLP: SMP_R.SMP_R.742 | 720575940631589407 | 9 |  | Murthy and Seung Labs: Austin T Burke (17), James Hebditch (2), Kendrick Joules Vinson (1). Jefferis Lab: Anjali Pandey (1). |
| 40 | SMP & SLP: SLPal1 | 720575940618140283 | 8 |  | Jefferis Lab: A. Javier (3). Murthy and Seung Labs: Nash Hadjerol (1), Ariel Dagohoy (1), James Hebditch (2). |
| 41 | SMP & SLP: SMPpd2 | 720575940619949556 | 7 |  | Jefferis Lab: Irene Salgarella (3), Anjali Pandey (1). |
| 42 | SMP & SLP: SMP_R.SMP_R.757 | 720575940632526547 | 6 |  | Murthy and Seung Labs: Austin T Burke (1), Kyle Patrick Willie (6), Mendell Lopez (10), Doug Bland (24), remer tancontian (6). Jefferis Lab: Anjali Pandey (1). |
| 43 | SMP & SLP: SMP_L.SMP_L.706 | 720575940629298679 | 6 |  | Murthy Lab: Lucas Encarnacion-Rivera (3). Jefferis Lab: A. Javier (1), Varun Sane (8), Arti Yadav (1). Murthy and Seung Labs: Kendrick Joules Vinson (7), remer tancontian (2). |
| 44 | SMP & SLP: SMP_R.SMP_R.906 | 720575940640456923 | 6 |  | Jefferis Lab: Philipp Schlegel (3), Yijie Yin (1), Varun Sane (6), Anjali Pandey (1). Selcho Lab: Mareike Selcho (17). Murthy and Seung Labs: Mendell Lopez (19), Kyle Patrick Willie (1). |
| 45 | SMP & SLP: DM3; right | 720575940621647498 | 5 |  | Jefferis Lab: A. Javier (1), Yijie Yin (1). |
| 46 | SMP & SLP: DM3_canonical | 720575940619899668 | 5 |  | Jefferis Lab: Yijie Yin (16). Murthy and Seung Labs: Nash Hadjerol (1), Kyle Patrick Willie (1). |
| 47 | SMP & SLP: SMP_R.SMP_R.677 | 720575940629901307 | 5 |  | Jefferis Lab: Yijie Yin (2), Chitra Nair (1), Varun Sane (3). Murthy and Seung Labs: Zairene Lenizo (2). |
| 48 | SMP & SLP: SLP_L.LH_L.5 | 720575940616707545 | 5 |  | Jefferis and Wilson Labs: Laia Serratosa Capdevila (1). Wes Murfin (4). Jefferis Lab: Yijie Yin (3), Varun Sane (1), Dhara Kakadiya (2), Chitra Nair (4). Murthy and Seung Labs: Doug Bland (3), Kendrick Joules Vinson (23), Joshua Bañez (10), Rey Adrian Candilada (1). |
| 49 | SMP & SLP: SLP_R.SLP_R.557 | 720575940629163419 | 5 |  | Murthy and Seung Labs: Austin T Burke (3). Jefferis Lab: Tomke S (14), Griffin Badalemente (4), Yijie Yin (1), Varun Sane (4), Dhwani Patel (1). |
| 50 | SMP & SLP: SMP_L.SMP_L.578 | 720575940626063688 | 5 | 270 | Murthy and Seung Labs: Austin T Burke (13). |
| 51 | CCHa2R (RA) | 720575940638190133 | 65 |  | Murthy and Seung Labs: Claire McKellar (1). Jefferis Lab: Anjali Pandey (1), Laia Serratosa (117), Dhwani Patel (3). Scott Lab: Amanda Abusaif (1), Zepeng Yao (27). Kim Lab: hanetwo (1). |
| 52 | CCHa2R (RA) | 720575940615181910 | 64 |  | Jefferis Lab: Anjali Pandey (1). Scott Lab: Amanda Abusaif (12). Murthy and Seung Labs: Michelle Pantujan (41), Ryan Willie (459). |
| 53 | CCHa2R (RA) | 720575940629642460 | 50 |  | Jefferis and Waddell Labs: Joseph Hsu (1). Murthy and Seung Labs: Claire McKellar (12), Ryan Willie (1), Shirleyjoy Serona (1), Doug Bland (296), J. Anthony Ocho (3). Jefferis Lab: Arti Yadav (1). Kim Lab: Hyungjun Choi (74). |
| 54 | CCHa2R (RA) | 720575940611411162 | 49 | 228 | Murthy and Seung Labs: Claire McKellar (2), Nash Hadjerol (2), Zairene Lenizo (1). Jefferis Lab: Irene Salgarella (3). Scott Lab: Amanda Abusaif (11). Kim Lab: hanetwo (14),Chan Hyuk Kang (2). |
| 55 | SMP & SEZ: ADM09 | 720575940628462927 | 48 |  | Anderson Lab: Altyn Rymbek (2). Jefferis Lab: Imaan Tamimi (6), Laia Serratosa (10), Rashmita Rana (1), Sangeeta Sisodiya (1). Murthy and Seung Labs: James Hebditch (1), Joshua Bañez (1), Mendell Lopez (58), Zairene Lenizo (2), J. Dolorosa (1). Kim Lab: Minsik Yun (1). |
| 56 | SMP & SEZ: pCd1? | 720575940618057095 | 22 |  | Jefferis Lab: A. Javier (1), Irene Salgarella (1). Murthy and Seung Labs: Austin T Burke (23) |
| 57 | SMP & SEZ: pCd1? | 720575940604332460 | 12 |  | Seung Lab: Zhihao Zheng (1). Jefferis and Wilson Labs: Laia Serratosa Capdevila (1). Jefferis: A. Javier (1), Bhargavi Parmar (1). Murthy and Seung Labs: Austin T Burke (13). |
| 58 | SMP & SEZ: ADM09p | 720575940642910152 | 12 |  | Scott Lab: Zepeng Yao (6). Murthy and Seung Labs: Austin T Burke (3), Shaina Mae Monungolh (1). Jefferis Lab: Yijie Yin (4), Marina Gkantia (10). |
| 59 | SMP & SEZ: DM3 | 720575940626005330 | 11 |  | Janelia tracers: Tansy Yang (5). Jefferis Lab: Yijie Yin (3). Murthy and Seung Labs: Austin T Burke (7). |
| 60 | SMP & SEZ: Dh44 | 720575940618579505 | 10 |  | Jefferis Lab: Imaan Tamimi (1), A. Javier (4), Rashmita Rana (1). Murthy and Seung Labs: Austin T Burke (13), remer tancontian (7). |
| 61 | SMP & SEZ: pCd1? | 720575940645882420 | 10 |  | Jefferis and Waddell Labs: Joseph Hsu (1). Murthy and Seung Labs: Austin T Burke (18), Joshua Bañez (1). |
| 62 | SMP & SEZ: pCd1? | 720575940638671219 | 10 |  | Jefferis Lab: A. Javier (3), Irene Salgarella (2), Tomke S (2), Varun Sane (5). Murthy and Seung Labs: Claire McKellar (2), remer tancontian (5), Nash Hadjerol (7). |
| 63 | SMP & SEZ: pCd1? | 720575940622119861 | 8 |  | Jefferis Lab: Irene Salgarella (2), Varun Sane (5), Yijie Yin (2). Murthy and Seung Labs: Austin T Burke (21). |
| 64 | SMP & SEZ: DM2_dorsal | 720575940629754588 | 8 |  | Anderson Lab: Altyn Rymbek (1). Jefferis Lab: Yijie Yin (11), Varun Sane (4). Murthy and Seung Labs: Austin T Burke (1), Joshua Bañez (1). |
| 65 | SMP & SEZ: SMPpv2 | 720575940621362044 | 7 |  | Jefferis and Wilson Labs: Laia Serratosa Capdevila (1). Jefferis Lab: Yijie Yin (34), Irene Salgarella (1), Imaan Tamimi (7), Chitra Nair (1). Murthy and Seung Labs: Shirleyjoy Serona (1), Austin T Burke (1). |
| 66 | SMP & SEZ: FLA_R.NO_OUT.6 | 720575940623184567 | 7 |  | Jefferis Lab: Katharina Eichler (1), Philipp Schlegel (1). Murthy and Seung Labs: Austin T Burke (1), Ben Silverman (2). |
| 67 | SMP & SEZ: pCd1? | 720575940638719907 | 7 |  | Jefferis and Waddell Labs: Joseph Hsu (1). Murthy and Seung Labs: Austin T Burke (1), Kyle Patrick Willie (6). Wes Murfin (29). |
| 68 | SMP & SEZ: pCd1? | 720575940612572054 | 6 |  | Seung Lab: Zhihao Zheng (1). Jefferis Lab: Irene Salgarella (6). Murthy and Seung Labs: Austin T Burke (37). |
| 69 | SMP & SEZ: DM2 | 720575940608174894 | 5 |  | Anderson Lab: Altyn Rymbek (1), Jefferis and Waddell Labs: Joseph Hsu (14). |
| 70 | SMP & SEZ: DM2 | 720575940635196334 | 5 |  | Jefferis Lab: Irene Salgarella (6). Janelia tracers: Tansy Yang (11). Murthy and Seung Labs: Austin T Burke (7). |
| 71 | SMP & SEZ: pMP5/DM2 | 720575940628732610 | 5 |  | Murthy and Seung Labs: Austin T Burke (4), remer tancontian (5). Jefferis Lab: Irene Salgarella (3), Yijie Yin (22). Janelia tracers: Tansy Yang (2). |
| 72 | SMP & SEZ: SMP_R.FLA_L.29 | 720575940636113264 | 5 |  | Jefferis Lab: Greg Jefferis (1), Laia Serratosa (11), Anjali Pandey (1). Dickson Lab: Alisa Poh (1). Murthy and Seung Labs: Nash Hadjerol (11). |
| 73 | SMP & SEZ: pCd1? | 720575940638285184 | 5 | 203 | Murthy and Seung Labs: Austin T Burke (3). Wes Murfin (9). Jefferis Lab: Laia Serratosa (4), Varun Sane (4). |
| 74 | Antler L | 720575940622998967 | 54 |  | Murthy and Seung Labs: Austin T Burke (17), Zairene Lenizo (4), Darrel Jay Akiatan (1), Shirleyjoy Serona (12), Shaina Mae Monungolh (2). Jefferis Lab: Yijie Yin (3). |
| 75 | Antler R | 720575940631226439 | 50 | 104 | Murthy and Seung Labs: Austin T Burke (17), Rey Adrian Candilada (3). Jefferis Lab: Varun Sane (7), Yijie Yin (5). |
| 76 | visual projection: SMP_R.SMP_R.201 | 720575940616608837 | 23 |  | Jefferis Lab: Irene Salgarella (1), Rashmita Rana (1), Griffin Badalemente (4). Murthy and Seung Labs: J. Anthony Ocho (12), J. Dolorosa (1). |
| 77 | visual projection: SMP_R.SMP_R.698 | 720575940630512711 | 17 |  | Jefferis Lab: Arti Yadav (1). Murthy and Seung Labs: Doug Bland (19). |
| 78 | visual projection: SMP_L.SMP_L.880 | 720575940636320063 | 15 |  | Murthy and Seung Labs: J. Dolorosa (26). Jefferis Lab: Rashmita Rana (1). |
| 79 | visual projection: SMP_L.SMP_L.362 | 720575940620022960 | 12 | 67 | Kim Lab: Dustin Garner (2). Jefferis Lab: Yijie Yin (16). Murthy and Seung Labs: Ben Silverman (5), remer tancontian (1). |
| 80 | Fan shaped body: DL1_dorsal; left | 720575940626612254 | 29 |  | Jefferis Lab: Rashmita Rana (1), Zeba Vohra (1). Murthy and Seung Labs: Austin T Burke (3), Kyle Patrick Willie (1). |
| 81 | Fan shaped body: FBl1-2 | 720575940642476448 | 17 |  | Murthy and Seung Labs: Jay Gager (1), James Hebditch (4), Kendrick Joules Vinson (1). Jefferis Lab: Varun Sane (6), Yijie Yin (3). |
| 82 | Fan shaped body: FB.FB.473 | 720575940619615936 | 7 |  | Jefferis Lab: Imaan Tamimi (1), A. Javier (1), Laia Serratosa (3), Arti Yadav (1), Sangeeta Sisodiya (7). |
| 83 | Fan shaped body: DL1 | 720575940613052200 | 5 | 58 | Jefferis Lab: Varun Sane (3), Griffin Badalemente (1). Murthy and Seung Labs: Shaina Mae Monungolh (1). |
| 84 | Gallinule | 720575940617054621 | 21 |  | Jefferis Lab: Anjali Pandey (1). Murthy and Seung Labs: Mendell Lopez (1), Joshua Bañez (2). |
| 85 | Gallinule | 720575940630349905 | 18 |  | Seeds Hampel Lab: Lucia Kmecova (1). Huetteroth Lab: Wolf Huetteroth (3). Jefferis Lab: Laia Serratosa (3). |
| 86 | Gallinule | 720575940629913130 | 7 |  | Murthy and Seung Labs: Zairene Lenizo (12). |
| 87 | Gallinule | 720575940620228449 | 5 |  | Jefferis Lab: Yijie Yin (1). Murthy and Seung Labs: remer tancontian (10), Zairene Lenizo (1). Itisha Joshi (4). |
| 88 | Gallinule | 720575940633711028 | 5 | 56 | Murthy and Seung Labs: Claire McKellar (6) |
| 89 | SEZ: PRW.PRW.213 | 720575940625172016 | 16 |  | Jefferis and Waddell Labs: Joseph Hsu (14). Murthy and Seung Labs: Darrel Jay Akiatan (31), Doug Bland (23), Austin T Burke (1), Ryan Willie (3), remer tancontian (1). |
| 90 | SEZ: PRW.GNG.43 | 720575940630664556 | 8 |  | Jefferis Lab: Marta Costa (1), Sangeeta Sisodiya (2). Seeds Hampel Lab: Alexis E Santana Cruz (2). Murthy and Seung Labs: Zairene Lenizo (3), Darrel Jay Akiatan (5), Ariel Dagohoy (1), Rey Adrian Candilada (2), Joshua Bañez (1), Nash Hadjerol (6), remer tancontian (1). |
| 91 | SEZ: PRW.PRW.312 | 720575940632055521 | 7 |  | Murthy and Seung Labs: J. Dolorosa (3), Shirleyjoy Serona (103). Jefferis Lab: Arti Yadav (1). |
| 92 | SEZ: PRW.PRW.82 | 720575940616860758 | 6 |  | Jefferis Lab: Arti Yadav (1). Murthy and Seung Labs: Joshua Bañez (7), Zairene Lenizo (3), Doug Bland (12), J. Dolorosa (2), remer tancontian (1). |
| 93 | SEZ: PRW.PRW.164 | 720575940622457579 | 6 | 43 | Jefferis Lab: Arti Yadav (1). Murthy and Seung Labs: Michelle Pantujan (2), Shirleyjoy Serona (3). |
|  |  |  |  | 1742 |  |
