# Supplementary material for: Hunger- and thirst-sensing neurons modulate a neuroendocrine network to coordinate sugar and water ingestion
Source: eLife. 2023 Sep 21;12:RP88143. doi: 10.7554/eLife.88143 (PMC10513480; doi:10.7554/eLife.88143)
Supplement: Supplementary file 3. — Number of synapses from ISN postsynaptic partners onto CCAP neurons, including the Flywire tracing contributions of different laboratories. [file elife-88143-supp3.docx]

| **Name** | **Flywire ID** | **synapses from BiT2 (720575940621662332)** | **synapses from VESa1 (720575940632951597)** | **synapses from CCHa2R-RA (720575940621942021)** | **synapses from CCHa2R-RA (720575940627765903)** | **synapses from Cowboy (720575940611730674)** | **Total synapses** | **Tracing contributions (number of edits)** |
| --- | --- | --- | --- | --- | --- | --- | --- | --- |
| CCAP | 720575940646160948 | 18 | 8 | 9 | 5 | 0 | 40 | Jefferis Lab: Greg Jefferis (15), Zeba Vohra (17), A. Javier (14), Siqi Fang (9), Varun Sane (6), Dhara Kakadiya (4). Jefferis and Wilson: Laia Serratosa Capdevila (1). Murthy and Seung Labs: Michelle Pantujan (1), Shaina Mae Monungolh (1), Rey Adrian Candilada (2), regine salem (1), Nash Hadjerol (1), Joshua Bañez (1). |
| CCAP | 720575940621148993 | 19 | 14 | 5 | 7 | 5 | 50 | Murthy and Seung Labs: Austin T Burke (5), Rey Adrian Candilada (1), J. Anthony Ocho (8), Nash Hadjerol (26), Joshua Bañez (4), Ryan Willie (2). Jefferis Lab: A. Javier (28), Imaan Tamimi (1), Katharina Eichler (11), Mendell Lopez (80). Jefferis and Wilson Labs: Laia Serratosa Capdevila (2), Varun Sane (1). Janelia tracers: Tansy Yang (1). |
| Total synapses per cell type |  | 37 | 22 | 14 | 12 | 5 | 90 |  |
