# Supplementary material for: Hunger- and thirst-sensing neurons modulate a neuroendocrine network to coordinate sugar and water ingestion
Source: eLife. 2023 Sep 21;12:RP88143. doi: 10.7554/eLife.88143 (PMC10513480; doi:10.7554/eLife.88143)
Supplement: Supplementary file 4. [file elife-88143-supp4.docx]

| **Figure** | **Short Genotype** | **Full Genotype** |
| --- | --- | --- |
| 1A | nSyb | w1118 ; UAS-dcr2/+ ; UAS-nSynaptobrevin RNAi (attP2)/+ |
| 1A | nSyb | w1118 ; UAS-dcr2/+; UAS-nSynaptobrevin (attP2)/VT011155-Gal4 (attP2) |
| 1A | TRH | w1118 ; UAS-dcr2/+ ; UAS-Trh RNAi (attP2)/+ |
| 1A | TRH | w1118 ; UAS-dcr2/+ ; UAS-Trh RNAi (attP2)/VT011155-Gal4 (attP2) |
| 1A | ChAT | w1118 ; UAS-dcr2/+ ; UAS- ChAT RNAi (attP2)/+ |
| 1A | ChAT | w1118 ; UAS-dcr2/+ ; UAS- ChAT RNAi (attP2)/VT011155-Gal4 (attP2) |
| 1A | TBH | w1118 ; UAS-dcr2/+ ; UAS-Tbh RNAi (attP2)/+ |
| 1A | TBH | w1118 ; UAS-dcr2/+ ; UAS-Tbh RNAi (attP2)/VT011155-Gal4 (attP2) |
| 1A | HDC | w1118 ; UAS-dcr2/+ ; UAS-Hdc RNAi (attP2)/+ |
| 1A | HDC | w1118 ; UAS-dcr2/+ ; UAS-Hdc RNAi (attP2)/VT011155-Gal4 (attP2) |
| 1A | VMAT | w1118 ; UAS-dcr2/+; UAS-VMAT RNAi (attP2)/+ |
| 1A | VMAT | w1118 ; UAS-dcr2/+ ; UAS-VMAT RNAi (attP2)/VT011155-Gal4 (attP2) |
| 1A | GAD1 | w1118 ; UAS-dcr2/+; UAS-GAD1 RNAi (attP2)/+ |
| 1A | GAD1 | w1118 ; UAS-dcr2/+ ; UAS-GAD1 RNAi (attP2)/VT011155-Gal4 (attP2) |
| 1A | DDC | w1118 ; UAS-dcr2/+ ; UAS-DDC RNAi (attP2)/+ |
| 1A | DDC | w1118 ; UAS-dcr2/+ ; UAS-DDC RNAi (attP2)/VT011155-Gal4 (attP2) |
| 1A | DVGlut | w1118 ; UAS-dcr2/+ ; UAS-DVGlut RNAi (attP2)/+ |
| 1A | DVGlut | w1118 ; UAS-dcr2/+ ; UAS-DVGlut RNAi (attP2)/VT011155-Gal4 (attP2) |
| 1A | sNPF | w1118 ; UAS-dcr2/+ ; UAS-sNPF RNAi (attP2)/+ |
| 1A | sNPF | w1118 ; UAS-dcr2/+ ; UAS-sNPF RNAi (attP2)/VT011155-Gal4 (attP2) |
| 1A | VGAT | w1118 ; UAS-dcr2/+ ; UAS-VGAT RNAi (attP2)/+ |
| 1A | VGAT | w1118 ; UAS-dcr2/+ ; UAS-VGAT RNAi (attP2)/VT011155-Gal4 (attP2) |
| 1A | TDC2 | w1118 ; UAS-dcr2/+ ; UAS-Tdc2 RNAi (attP2)/+ |
| 1A | TDC2 | w1118 ; UAS-dcr2/+ ; UAS-Tdc2 RNAi (attP2)/VT011155-Gal4 (attP2) |
| 1A | dILP1 | w1118 ; UAS-dcr2/+ ; UAS-dILP1 RNAi (attP2)/+ |
| 1A | dILP1 | w1118 ; UAS-dcr2/+ ; UAS-dILP1 RNAi (attP2)/VT011155-Gal4 (attP2) |
| 1A | dILP2 | w1118 ; UAS-dcr2/+ ; UAS-dILP2 RNAi (attP2)/+ |
| 1A | dILP2 | w1118 ; UAS-dcr2/+ ; UAS-dILP2 RNAi (attP2)/VT011155-Gal4 (attP2) |
| 1A | dILP3 | w1118 ; UAS-dcr2/+ ; UAS-dILP3 RNAi (attP2)/+ |
| 1A | dILP3 | w1118 ; UAS-dcr2/+ ; UAS-dILP3 RNAi (attP2)/VT011155-Gal4 (attP2) |
| 1A | dILP4 | w1118 ; UAS-dcr2/+ ; UAS-dILP4 RNAi (attP2)/+ |
| 1A | dILP4 | w1118 ; UAS-dcr2/+ ; UAS-dILP4 RNAi (attP2)/VT011155-Gal4 (attP2) |
| 1A | dILP5 | w1118 ; UAS-dcr2/+ ; UAS-dILP5 RNAi (attP2)/+ |
| 1A | dILP5 | w1118 ; UAS-dcr2/+ ; UAS-dILP5 RNAi (attP2)/VT011155-Gal4 (attP2) |
| 1A | dILP6 | w1118 ; UAS-dcr2/+ ; UAS-dILP6 RNAi (attP2)/+ |
| 1A | dILP6 | w1118 ; UAS-dcr2/+ ; UAS-dILP6 RNAi (attP2)/VT011155-Gal4 (attP2) |
| 1A | dILP7 | w1118 ; UAS-dcr2/+ ; UAS-dILP7 RNAi (attP2)/+ |
| 1A | dILP7 | w1118 ; UAS-dcr2/+ ; UAS-dILP7 RNAi (attP2)/VT011155-Gal4 (attP2) |
| 1B | dILP3 RNAi | w1118 ; + ; UAS-dILP3 RNAi (attP2)/+ |
| 1B | dILP3 RNAi | w1118 ; + ; UAS-dILP3 RNAi (attP2)/VT011155-Gal4 (attP2) |
| 1B | Amon RNAi | w1118/w* ; + ; UAS-amon RNAi (attP2)/+ |
| 1B | Amon RNAi | w1118/w* ; + ; UAS-amon RNAi (attP2)/VT011155-Gal4 (attP2) |
| 1B | ISN-Gal4 | w1118 ; + ; VT011155-Gal4 (attP2)/+ |
| 1D | ISN-Gal4 > tdT | w1118/w* ; UAS-myrGFP.QUAS-mtdTomato-3xHA/+; trans-Tango/VT011155-Gal4 (attP2) |
| 2B,2F | BiT split-Gal4 > Chrimson | w1118, UAS-IVS-CsChrimson.mVenus (attP18)/+; VT002073-Gal4.AD (attP40)/+; VT040568-Gal4.DBD (attP2)/+ |
| 2D, 2E | ISN > Chrimson, BiT > ArcLight | 13XLexAop2-IVS-p10-ChrimsonR-mCherry (attP18)/w1118; GMR34G02-LexA (attP40)/VT002073-Gal4.AD (attP40); VT040568-Gal4.DBD (attP2)/UAS-ArcLight (attP2) |
| 2F, 2G | BiT split-Gal4 | w1118; VT002073-Gal4.AD (attP40)/+; VT040568-Gal4.DBD (attP2)/+ |
| 2F | Empty split-Gal4 > Chrimson | w1118, UAS-IVS-CsChrimson.mVenus (attP18); p65-AD.empty (attp40)/+; GAL4-DBD.empty (attP2)/+ |
| 2F | Gr5a-Gal4 > Chrimson | w1118, UAS-IVS-CsChrimson.mVenus (attP18); Gr5a-Gal4/+; Gr5a-Gal4/+ |
| 2F | ppk28-Gal4 > Chrimson | w1118, UAS-IVS-CsChrimson.mVenus (attP18); + ; ppk28-Gal4/+ |
| 2G | BiT split-Gal4 > nSyb RNAi | w1118; VT002073-Gal4.AD (attP40)/+; VT040568-Gal4.DBD (attP2)/UAS-nSynaptobrevin RNAi (attP2) |
| 2G, 4G, 5G | nSyb RNAi | w1118; +; UAS-nSynaptobrevin RNAi (attP2)/+ |
| 4B, 4F | CCHa2R (RA) > Chrimson | w1118, UAS-IVS-CsChrimson.mVenus (attP18)/w1118; TI{2A-GAL4}CCHa2-R[2A-A.GAL4]/+; + |
| 4D, 4E | ISN > Chrimson, CCHa2R (RA) > GCaMP | w1118; GMR34G02-LexA (attP40)/ TI{2A-GAL4}CCHa2-R[2A-A.GAL4]; LexAop-Chrimson,UAS-GCaMP6s/TM2 |
| 4F, 4G | CCHa2R (RA) | w1118; TI{2A-GAL4}CCHa2-R[2A-A.GAL4]/+; + |
| 4F, 5F | UAS-Chrimson | w1118, UAS-IVS-CsChrimson.mVenus (attP18)/w1118; +; + |
| 4G | CCHa2R (RA) > nSyb RNAi | w1118; TI{2A-GAL4}CCHa2-R[2A-A.GAL4]/+; UAS-nSynaptobrevin RNAi (attP2)/+ |
| 5B, 5F | CCAP > Chrimson | w1118, UAS-IVS-CsChrimson.mVenus (attP18)/w1118; CCAP-Gal4/+; + |
| 5D, 5E | ISN > Chrimson, CCAP > GCaMP | w1118, 13XLexAop2-IVS-p10-ChrimsonR-mCherry (attP18)/w1118; GMR34G02-LexA (attP40)/CCAP-Gal4; 20XUAS-IVS-jGCaMP7b(VK00005)/+ |
| 5F, 5G | CCAP | w1118; CCAP-Gal4/+; + |
| 5G | CCAP > NSyb RNAi | w1118; CCAP-Gal4/+; UAS-nSynaptobrevin RNAi (attP2)/+ |
| Supp 1A | ISN > transTango | w*/w1118 ; UAS-myrGFP.QUAS-mtdTomato-3xHA/+; trans-Tango/VT011155-Gal4 (attP2) |
| Supp 2A,B | BiT genetic control | w1118, 13XLexAop2-IVS-p10-ChrimsonR-mCherry (attP18)/w1118; VT002073-Gal4.AD (attP40)/+; VT040568-Gal4.DBD (attP2)/UAS-ArcLight (attP2) |
| Supp 3A,B | BiT > Chrimson, IPC > GCaMP | 10XUAS-syn21-Chrimson88-tdT3.1 (attP18), LexAop2-syn21-opGCaMP6s suHw (attP8)/w1118; TI{2A-lexA::GAD}CCHa2-R[2A-A.lexA]/VT002073-Gal4.AD (attP40); VT040568-Gal4.DBD (attP2)/+ |
| Supp 3C,D | IPC genetic control | 10XUAS-syn21-Chrimson88-tdT3.1 (attP18), LexAop2-syn21-opGCaMP6s suHw (attP8)/w1118; dILP2-LexA/CyO; TM2/TM3, Sb |
| Supp 4A,B | CCHa2R (RA) genetic control | w1118; TI{2A-GAL4}CCHa2-R[2A-A.GAL4]/CyO; LexAop-Chrimson,UAS-GCaMP6s/TM2 |
| Supp 4C,D | BiT > Chrimson, CCHa2R (RA) > GCaMP | 10XUAS-syn21-Chrimson88-tdT3.1 (attP18), LexAop2-syn21-opGCaMP6s suHw (attP8)/w1118; TI{2A-lexA::GAD}CCHa2-R[2A-A.lexA]/VT002073-Gal4.AD (attP40); VT040568-Gal4.DBD (attP2)/+ |
| Supp 5 | ISN > Chrimson,  CCAP > GCaMP | 13XLexAop2-IVS-p10-ChrimsonR-mCherry (attP18)/w1118; GMR34G02-LexA (attP40)/+ ; 20XUAS-IVS-jGCaMP7b(VK00005)/CCAP-Gal4 |
